# A Study on Contraceptive Choices and Usage Trends Among Reproductive Age Individuals in Urban and Rural Areas

**DOI:** 10.7759/cureus.86721

**Published:** 2025-06-25

**Authors:** Ishita Rathore, Logeswari B M, Niveditha Prasath

**Affiliations:** 1 Obstetrics and Gynaecology, Sree Balaji Medical College and Hospital, Chennai, IND

**Keywords:** contraceptive agents, family planning practices, health seeking behavior, reproductive health, urban and rural communities

## Abstract

Background

Contraceptive utilization is a key element of reproductive health and family planning. Despite national initiatives promoting modern contraceptive methods in India, substantial disparities persist between urban and rural populations. This study was done to compare the prevalence, types, and determinants of contraceptive use, including unmet needs, among reproductive-aged individuals in urban and rural settings.

Methods

A mixed-methods study was conducted over three months among 1,000 individuals (608 urban, 392 rural) aged ≥18 years in Chennai, Tamil Nadu. Participants were recruited through convenience sampling from urban and rural health training centers affiliated with Sree Balaji Medical College and Hospital. Data were collected using a structured questionnaire covering demographics, reproductive history, contraceptive use, and reasons for unmet needs. Quantitative data were analyzed using SPSS software, version 23 (IBM Corp., Armonk, NY) with Chi-square tests and logistic regression; qualitative data underwent thematic analysis.

Results

The overall contraceptive prevalence was 58.7%, significantly higher in urban areas (74.01%) than rural areas (34.95%) (p < 0.001). Female sterilization was most common in rural areas (50%), while urban participants favored barrier methods (25%) and oral contraceptive pills (14.97%). Literacy (adjusted odds ratio (AOR) = 2.80; 95% CI: 2.05-3.82) and urban residence (AOR = 4.20; 95% CI: 3.25-5.43) were significant predictors. Barriers included fear of side effects, religious beliefs, spousal opposition, and limited service access.

Conclusion

This study highlights a stark contrast in contraceptive trends between urban and rural populations, shaped by educational status, access, cultural beliefs, and gender dynamics. Addressing unmet needs in family planning requires community-tailored interventions focusing on male engagement, contraceptive education, and strengthening the health system to improve accessibility and autonomy in reproductive choices.

## Introduction

Contraceptive use is a fundamental pillar of reproductive health and profoundly influences maternal and child health outcomes, population dynamics, and socioeconomic development. Globally, family planning initiatives are recognized as essential strategies for reducing maternal mortality, preventing high-risk pregnancies, and empowering individuals through informed reproductive choices [1]. In India, a nation characterized by diverse socio-cultural contexts and significant urban-rural disparities, the prevalence and patterns of contraceptive use reflect a complex interplay of demographic, economic, and cultural determinants [2]. Despite notable progress in expanding access to family planning services, challenges such as the unmet need for contraception, regional variations, and socio-demographic barriers persist, necessitating targeted research to inform evidence-based policy and practice [3].

The burden of inadequate contraceptive use in India is also considerable. National estimates indicate that approximately 58% of married women aged 15-49 years employ some form of contraception, yet significant gaps remain, particularly in rural areas and among younger populations [4]. Traditional methods, such as withdrawal and periodic abstinence, continue to be prevalent in certain communities, often because of limited access to modern contraceptives or entrenched cultural preferences [5]. Furthermore, the unmet need for family planning, defined as the proportion of fecund, sexually active women who wish to limit or space births but do not use contraception, remains a critical concern. Studies suggest that 12-15% of married women in India experience unmet needs, with higher rates in states such as Bihar and Uttar Pradesh [6]. This unmet need contributes to unintended pregnancies, unsafe abortions, and increased maternal and infant mortality, underscoring the urgency of addressing barriers to contraceptive access and uptake [7].

Trends in contraceptive use in India have revealed both advancements and persistent challenges. In recent decades, the contraceptive prevalence rate has increased, driven by government initiatives such as the National Family Planning Programme and campaigns promoting modern reversible methods such as intrauterine devices (IUDs) and oral contraceptives [8]. However, sterilization, particularly female sterilization, continues to dominate the contraceptive method mix, accounting for nearly 75% of modern methods used in some regions [9]. This reliance on permanent methods reflects systemic issues, including the limited availability of reversible options, inadequate counseling, and socio-cultural pressures, notably from older family members such as mothers-in-law [10]. Additionally, urban-rural disparities are pronounced, with urban women having greater access to a diverse range of contraceptives than their rural counterparts, who often face logistical barriers and lower health literacy [11]. Younger women, particularly those aged 15-24 years living in urban slums, exhibit higher unmet needs due to socioeconomic constraints and limited autonomy in reproductive decision-making [12].

This study was undertaken to generate detailed, context-specific data on contraceptive usage and its determinants across urban and rural settings in Chennai. While national surveys like the National Family Health Survey (NFHS) and District Level Household and Facility Survey (DLHS) provide broad insights, they often lack the granularity to explore local variations and sociocultural influences on contraceptive choices. Cultural and religious beliefs, along with social dynamics, such as the influence of older family members, play a critical role in shaping reproductive decisions, yet remain underexplored in large-scale datasets. This study addresses these gaps by including both men and women aged ≥18 years, thereby acknowledging the role of male partners in family planning. The use of a cross-sectional design ensured representation across diverse socioeconomic and educational backgrounds, enhancing the relevance of findings within the study area. The inclusion of qualitative interviews further enhances the study’s ability to capture personal narratives and cultural nuances, which are often overlooked in statistical analyses. Conducted through urban and rural health training centres linked to medical institutions, the study bridges public health research with clinical perspectives. The primary objective was to compare the prevalence, types, and determinants of contraceptive use, including unmet needs, between urban and rural populations, along with the qualitative aspects.

## Materials and methods

Study design and setting

This study was a community-based, mixed-methods study with a cross-sectional survey as a quantitative part conducted over a period of three months. The research was conducted in urban and rural field practice areas affiliated with the Department of Community Medicine, Sree Balaji Medical College and Hospital (SBMCH), Chennai, between January and May 2025. Urban data collection was conducted at the Urban Health Training Centre (UHTC), Anakaputhur, and rural data were gathered from the Rural Health Training Centre (RHTC), Padapai. These centers serve diverse populations and are integral to community health training and service delivery. Additionally, to enhance the diversity of participants and ensure the inclusion of a broader range of sociodemographic characteristics, data were also collected from the Department of Obstetrics and Gynecology at SBMCH. This allowed for the inclusion of individuals seeking reproductive health services within a clinical setting, thereby providing a more comprehensive representation of contraceptive usage in both field and institutional contexts.

Study population and eligibility criteria

The study population included men and women aged 18 years and above who were permanent residents of the selected urban or rural field areas. These individuals were considered to be of reproductive age and potentially involved in making or influencing contraceptive decisions either directly or indirectly. Recruitment was conducted during outpatient visits and community outreach activities organized by the health centers.

Inclusion and exclusion criteria

Participants eligible for inclusion in the study were adults aged 18 years and above of either sex, who were permanent residents of the selected urban or rural areas and had provided written informed consent to participate. To maintain the focus on active contraceptive decision-making, certain groups were excluded from the study. These included individuals under 18 years of age, pregnant or postmenopausal women, and those who had already undergone permanent sterilization procedures such as tubectomy or vasectomy. Additionally, individuals identifying as non-binary or with unspecified gender identity were excluded, as the study centered on traditional gender roles in contraceptive behavior. Participants who lacked digital literacy or smartphone access and were therefore unable to participate in follow-up via WhatsApp, Facebook, or Instagram were also excluded.

Sample size determination

The minimum sample size for the study was calculated using the standard formula for estimating proportions:

n=Z2×P×Q/d2

Where: Z=1.96 (for a 95% confidence level), P=58% (estimated prevalence of contraceptive use from previous study) [13], Q=(100−P)=42%, d=5%(absolute precision). Substituting the values into the formula yielded a required sample size of approximately 375 participants with 80% power and a 95% Confidence Interval. To ensure large representation, 608 individuals were recruited from urban areas and 392 from rural areas, providing adequate power for urban-rural comparative analysis with a total of 1000 participants.

Sampling method

A convenience sampling technique was employed due to logistical and time constraints. Participants were approached during routine outpatient visits at health facilities and during community outreach activities carried out by healthcare workers. While convenience sampling may introduce selection bias, the research team took deliberate steps to enhance representativeness. Participants were selected to reflect a diverse range of ages, educational backgrounds, occupational statuses, and socioeconomic levels. Additionally, recruitment quotas were maintained across urban and rural locations to ensure balanced representation of both populations.

Data collection tools and techniques

Data were gathered using a pretested, structured questionnaire (see Appendix), developed in both English and Tamil to ensure accessibility and cultural relevance. The instrument was constructed following an extensive literature review and expert consultation, ensuring its validity and contextual appropriateness. The questionnaire comprised four key sections: (a) Sociodemographic profile - this section collected data on age, gender, marital status, education level, occupation, income, religion, and area of residence (urban/rural), (b) Reproductive and obstetric history - Information included age at marriage, number of pregnancies, parity, and number of living children, (c) Contraceptive practices - questions covered current and previous contraceptive use, preferred methods, duration of use, and who primarily made contraceptive decisions (self, spouse, or jointly), (d) Unmet need and barriers - this section explored reasons for non-use, including perceived side effects, cultural or religious beliefs, partner opposition, and access-related issues.

The tool incorporated both closed-ended questions (e.g., multiple-choice and Likert scales) to facilitate quantitative analysis and open-ended questions to capture individual experiences and context-specific responses. Data collection was conducted by trained medical interns and faculty members under the close supervision of the principal investigators. Interviews were conducted in private settings within health centers or during scheduled home visits, ensuring participant comfort and confidentiality. Every effort was made to maintain ethical standards and ensure the accuracy and integrity of the information collected. To supplement the quantitative findings, in-depth interviews ( see Appendix for the questionnaire) were conducted with a subset of participants, focusing on their personal beliefs, cultural norms, and perceived barriers to contraceptive access and usage.

Ethical considerations

The study received ethical approval from the Institutional Human Ethics Committee of Sree Balaji Medical College and Hospital, Chennai (002/SBMCH/IHEC/2025/2387). Participants were provided with a detailed information sheet and gave written informed consent before participation. The purpose, risks, and benefits of the study were explained in the participants' native language. No identifiable personal information was recorded, and participants were assured of the confidentiality of their responses. Participation was entirely voluntary, and refusal to participate did not affect the provision of healthcare services.

Statistical analysis

All quantitative data were entered into Microsoft Excel 2019 and analyzed using SPSS Software, version 23 (IBM Corp., Armonk, NY). Descriptive statistics such as frequencies, percentages, means, and standard deviations were calculated to summarize demographic and behavioral data. To identify independent predictors of contraceptive use, binary logistic regression was conducted. Adjusted odds ratios (AORs) with 95% confidence intervals (CIs) were reported. A p-value < 0.05 was considered statistically significant. For qualitative data, a thematic analysis approach was used. Transcripts from in-depth interviews were coded inductively to identify emerging patterns related to contraceptive decision making, cultural attitudes, and health system barriers.

## Results

Table 1 presents the demographic distribution of the study population (N = 1000), comprising 608 urban and 392 rural participants. Females accounted for a higher proportion overall (56.9%), with 57.4% from urban and 56.1% from rural areas. The majority of participants were aged 18-36 years (54.3%), with a higher proportion in urban areas (58.4%) compared to rural areas (48%). Notably, 30.8% of the total sample were females aged 18-36 years, while 26.1% were females aged over 37 years. A significant literacy gap was observed, with 89.97% of urban and only 69.9% of rural participants being literate. Regarding occupation, daily wage laborers were more prevalent in rural areas (50%) compared to urban areas (20%), while formal employment was more common in urban participants (40%). Socioeconomic status showed a clear urban-rural divide: 45.07% of urban residents belonged to higher socioeconomic status (SES) classes (Class I and II), while 65.05% of rural participants fell into lower SES categories (Class IV and V), highlighting economic disparities between the two populations.

**Table 1:** Distribution of socio-demographic variables among the study participants (n=1000) *Modified BG Prasad scale 2025 [14].

Variable	Category	Urban n (%)	Rural n (%)	Total n (%)
Gender	Male	259 (42.60%)	172 (43.88%)	431 (43.10%)
	Female	349 (57.40%)	220 (56.12%)	569 (56.90%)
Age Group (years)	18–36	355 (58.39%)	188 (47.96%)	543 (54.30%)
	>37	253 (41.61%)	204 (52.04%)	457 (45.70%)
Age & Gender	18–36, Male	142 (23.36%)	93 (23.72%)	235 (23.50%)
	18–36, Female	213 (35.03%)	95 (24.23%)	308 (30.80%)
	>37, Male	117 (19.24%)	79 (20.15%)	196 (19.60%)
	>37, Female	136 (22.37%)	125 (31.89%)	261 (26.10%)
Education	Illiterate	61 (10.03%)	118 (30.10%)	179 (17.90%)
	Literate	547 (89.97%)	274 (69.90%)	821 (82.10%)
Occupation	Daily Wage Labourer	122 (20.07%)	196 (50.00%)	318 (31.80%)
	Employed	243 (39.97%)	78 (19.90%)	321 (32.10%)
	Homemaker	243 (39.97%)	118 (30.10%)	361 (36.10%)
Socioeconomic Status (SES)*	Class I	122 (20.07%)	20 (5.10%)	142 (14.20%)
	Class II	152 (25.00%)	39 (9.95%)	191 (19.10%)
	Class III	182 (29.93%)	78 (19.90%)	260 (26.00%)
	Class IV	91 (14.97%)	137 (34.95%)	228 (22.80%)
	Class V	61 (10.03%)	118 (30.10%)	179 (17.90%)

Table 2 outlines the reproductive characteristics of the total study population (N = 1000), comprising 608 urban and 392 rural participants. Regarding parity, nulliparous individuals were more common in urban areas (182; 29.93%) than in rural areas (59; 15.05%), while participants with ≥2 children were significantly higher in rural areas (235; 59.95%) compared to urban (183; 30.10%). Participants with one child comprised 243 (39.97%) in urban and 98 (25.00%) in rural settings. When asked about planning the current pregnancy, only 122 (20.07%) of urban participants responded "yes" compared to 137 (34.95%) in the rural group. A majority in both settings reported unplanned pregnancies, particularly in urban areas (486; 79.93%) vs. rural (255; 65.05%). The number of living children followed a similar pattern: 0 children was reported by 195 urban (32.07%) and 71 rural (18.11%) participants; one child by 231 urban (37.99%) and 86 rural (21.94%); and ≥2 children by 182 urban (29.93%) and 235 rural (59.95%). With regard to age at first pregnancy, urban participants were more likely to have conceived later, with 243 (39.97%) between 26-30 years and 122 (20.07%) after 30 years, compared to rural participants, where 157 (40.05%) reported first pregnancies at 20-25 years and 118 (30.10%) before age 20. These trends highlight stark differences in fertility patterns, pregnancy planning, and reproductive timing between urban and rural populations.

**Table 2:** Obstetric characteristics and current pregnancy intent among the study participants (n=1000)

Variables	Urban n (%) n=608	Rural n (%) n=392	Total (n)
Parity			
Nulliparous	182 (29.93)	59 (15.05)	241
1	243 (39.97)	98 (25.00)	341
≥2	183 (30.10)	235 (59.95)	418
Planning pregnancy			
Yes	122 (20.07)	137 (34.95)	259
No	486 (79.93)	255 (65.05)	741
Number of living children			
0	195 (32.07)	71 (18.11)	266
1	231 (37.99)	86 (21.94)	317
≥2	182 (29.93)	235 (59.95)	417
Age at first pregnancy (years)			
<20	61 (10.03)	118 (30.10)	179
20–25	182 (29.93)	157 (40.05)	339
26–30	243 (39.97)	78 (19.90)	321
>30	122 (20.07)	39 (9.95)	161

Table 3 presents the distribution of contraceptive method usage among 1,000 participants-608 urban and 392 rural. A clear urban-rural contrast is observed in method preference. In urban areas, the most commonly used method was the barrier method (condom), reported by 152 participants (25.00%), followed by oral contraceptive pills (OCPs) at 91 (14.97%), and sterilization at 122 (20.07%). In contrast, sterilization (tubectomy or vasectomy) was the most prevalent method in rural areas, used by 196 participants (50.00%), highlighting a greater reliance on permanent methods. Usage of injectable contraceptives was low in both groups (urban: 30; 4.93%, rural: 12; 3.06%), and intrauterine contraceptive devices (IUCDs) were used by 61 (10.03%) in urban and 20 (5.10%) in rural areas. Contraceptive implants had the lowest uptake overall, with 18 (2.96%) in urban and four (1.02%) in rural populations. The category labeled “others”-which may include natural methods, emergency contraception, or traditional practices-was reported by 134 urban (22.04%) and 101 rural (25.77%) participants.

**Table 3:** Types of contraceptive methods used among the study participants (n=1000) OCP: Oral contraceptive pills, IUCD: Intrauterine contraceptive device.

Contraceptive Method	Urban n (%) n=608	Rural n (%) n=392
Barrier (Condom)	152 (25.00)	39 (9.95)
OCP	91 (14.97)	20 (5.10)
Injectable	30 (4.93)	12 (3.06)
IUCD	61 (10.03)	20 (5.10)
Contraceptive implants	18 (2.96)	4 (1.02)
Sterilization (Tubectomy/Vasectomy)	122 (20.07)	196 (50.00)
Others	134 (22.04)	101 (25.77)

Table 4 presents the results of univariate and multivariate analyses exploring factors associated with contraceptive use among 1,000 participants, of whom 587 (58.7%) reported using contraception and 413 (41.3%) did not. Urban residence was strongly associated with contraceptive use, with 450 urban participants (74.01%) using contraception compared to only 137 rural participants (34.95%). Rural residents had significantly higher odds of non-use (adjusted OR (aOR)= 4.20, 95% CI: 3.25-5.43, p < 0.001). Education also played a key role: 527 literate individuals (64.19%) used contraception versus only 60 illiterate individuals (33.52%), with illiteracy associated with a higher likelihood of non-use (aOR = 2.80, 95% CI: 2.05-3.82, p < 0.001). Decision-making autonomy significantly influenced contraceptive use. Participants who made decisions themselves had the highest usage (260; 65.00%), whereas those whose spouse was the primary decision-maker had significantly lower usage (135; 45.00%), with an aOR of 1.90 (95% CI: 1.42-2.54). Joint decision-making did not show a statistically significant association (aOR = 1.02, 95% CI: 0.77-1.35, p = 0.198). Religion showed a non-significant trend: contraceptive use was reported by 64% of Christians/Others, 60% of Hindus, and 50% of Muslims. Compared to Christians/Others, Muslims had a higher odds of non-use (aOR = 1.50, 95% CI: 0.81-2.78, p = 0.198), but this was not statistically significant. 

**Table 4:** Associations between sociodemographic factors and contraceptive use or unmet need among participants (n=1000) OR: Odds ratio. Logistic regression; p-value<0.05 is statistically significant.

Variables	Contraception Use n (%) (n=587)	Contraception Not Used n (%) (n=413)	Unadjusted OR (95% CI)	p-value	Adjusted OR (95% CI)	p-value
Area of Residence						
Urban	450 (74.01)	158 (25.99)	Ref	<0.001	Ref	<0.001
Rural	137 (34.95)	255 (65.05)	5.30 (4.13, 6.80)		4.20 (3.25,5.43)	
Education						
Literate	527 (64.19)	294 (35.81)	Ref	<0.001	Ref	<0.001
Illiterate	60 (33.52)	119 (66.48)	3.55 (2.65, 4.75)		2.80 (2.05,3.82)	
Decision Maker						
Self	260 (65.00)	140 (35.00)	Ref	<0.001	Ref	
Spouse	135 (45.00)	165 (55.00)	2.27 (1.73, 2.97)		1.90 (1.42-2.54)	0.892
Joint	192 (64.00)	108 (36.00)	1.04 (0.79, 1.37)		1.02 (0.77,1.35)	0.198
Religion						
Christian/Others	32 (64.00)	18 (36.00)	Ref	0.015	Ref	
Hindu	480 (60.00)	320 (40.00)	1.19 (0.70, 2.03)		1.10 (0.63,1.92)	0.735
Muslim	75 (50.00)	75 (50.00)	1.78 (0.98, 3.23)		1.50 (0.81,2.78)	0.198

The thematic analysis represented in Table 5 highlights six key themes influencing contraceptive behavior among 40 participants, supported by representative quotations. Decision-making autonomy varied, with rural women frequently reporting spousal control, while urban participants described self or joint decision-making. Cultural and religious influences were strong in rural areas, where contraception was often seen as interfering with divine will. Awareness and health literacy showed rural-urban differences, with rural participants citing misconceptions and limited reproductive education, while urban respondents referenced media awareness but lacked detailed understanding. Accessibility and infrastructure barriers were more pronounced in rural settings, where stockouts and travel distances hindered access, unlike urban areas with better service availability. Male involvement and gender roles revealed limited male participation and resistance to vasectomy in rural areas, reinforcing contraception as a female responsibility. Lastly, method preferences and perceived risks showed rural preference for sterilization and urban inclination toward temporary methods, driven by fears of side effects and cultural norms. These findings underscore the complex interplay of social, cultural, and systemic factors shaping contraceptive choices.

**Table 5:** Thematic analysis of in-depth interviews on contraceptive use among urban and rural men and women (n = 40)

Theme	Subthemes	Representative Participant Quotations and Approximate Frequency
1. Decision-Making Autonomy	– Spousal or family-dominated decisions – Joint or self-initiated choices – Influence of marital hierarchy	“My husband said no to pills, so I didn’t take them.” – Rural Female (frequently mentioned by rural women) “I chose to use condoms; no one else influenced my decision.” – Urban Male (shared by several urban men and women)
2. Cultural and Religious Influences	– Religious prohibitions – Fatalistic outlook – Contraception as taboo	“We don’t interfere with God’s plan. Children are His blessing.”– Rural Male (expressed by many rural participants) “My religion doesn’t allow these artificial methods.” – Rural Female (repeated by both genders in rural settings)
3. Awareness and Health Literacy	– Misconceptions about side effects – Role of ASHA workers and media – Uneven access to reproductive education	“We thought injections make you permanently infertile.” – Rural Female (a common fear among less-educated rural women) “I heard from TV about copper-T but didn’t know how it works.”– Urban Female (mentioned by several urban participants)
4. Accessibility and Infrastructure Barriers	– Limited method availability – Travel burdens – Stock-outs at government centres	“There were no pills at the PHC this month, so I stopped using them.” – Rural Female (frequently reported in rural group) “Everything is available nearby in the city clinic.” – Urban Female (noted by most urban women)
5. Male Involvement and Gender Roles	– Low male participation – Resistance to vasectomy – Perception of contraception as a female domain	“They say men shouldn’t get operated; it weakens them.” – Rural Male (shared by most rural men) “He refused vasectomy, so I had to go for sterilization.” – Rural Female (a recurrent concern among rural women)
6. Method Preferences and Perceived Risks	– Permanent vs. temporary method bias – Preference for traditional/rhythm methods – Side-effect-related fears	“I got sterilized after two children—everyone does that here.” – Rural Female (widely normalized in rural interviews) “I’m afraid of pills; I prefer condoms or safe days.” – Urban Female (expressed by several educated urban users)

## Discussion

This study assessed contraceptive usage trends and their determinants among reproductive-aged individuals in both urban and rural populations. The findings demonstrated that contraceptive prevalence was significantly higher in urban areas (74.01%) than in rural areas (34.95%), indicating wide disparities in access, education, and decision-making autonomy. These findings are consistent with earlier reports by Srikanthan and Reid, who emphasized that religious and cultural contexts heavily influence contraceptive behavior, especially in less urbanized settings, where traditional norms are dominant and often restrict modern contraceptive uptake [15]. Our results also established a strong association between literacy and contraceptive use. Among the literate participants, 64.19% reported current contraceptive use, while usage dropped to 33.52% among illiterate individuals. This aligns with Anukriti et al., who reported that education substantially increases women’s autonomy and fertility choices, leading to a higher usage of reversible contraceptive methods [16].

Sterilization emerged as the most commonly used method, particularly in rural areas where 50% of participants had undergone permanent sterilization, compared to 20.07% in urban zones. Mozumdar et al. highlighted that India's family planning programs have historically emphasized female sterilization as the primary method, especially in lower-income and rural communities, owing to the limited availability of reversible options and provider bias [12]. Our study substantiates this, as many rural women preferred sterilization once family size goals were achieved, indicating both systemic gaps and entrenched sociocultural norms.

Additionally, urban respondents favored reversible methods, such as condoms (25%), oral contraceptive pills (14.97%), and intrauterine contraceptive devices (10.03%), reflecting a wider method choice and greater autonomy. Dutta et al. also observed a transition toward modern spacing methods in urban and educated populations driven by increasing awareness and health-seeking behaviors [17].

Socioeconomic status (SES) shows a distinct pattern in contraceptive choice. Urban individuals in higher SES classes reported better access and a broader method mix, whereas participants from lower SES classes, particularly in rural areas, relied on permanent methods. Sharma et al. corroborated this trend, reporting that SES strongly influences both the decision-making process and accessibility of contraception, with higher SES groups more likely to use modern spacing methods [18].

Decision-making autonomy significantly influences contraceptive use. In our study, individuals who were self-decision-makers had higher contraceptive usage (65%) than those where the spouse was the primary decision-maker (45%). NFHS data also support this, stating that a lack of decision-making power, particularly among women in patriarchal households, limits the uptake of temporary methods [19].

Traditional method use, while limited in our cohort, continues to persist in specific cultural and religious subsets. Johnson-Hanks pointed out that for many users, traditional methods are embedded in social timing and norms, rather than biomedical rationality, thereby affecting their contraceptive behavior [20]. The use of 'other methods' in our study, which was reported by 22.04% in urban and 25.77% in rural areas, possibly included traditional approaches or rhythm-based methods, underscoring the need for further exploration of the motivations behind such choices.

Concerns over side effects, costs, and service accessibility were frequently cited reasons for non-use. Russo et al. noted that fear of side effects and prior negative experiences remain strong deterrents for many women considering hormonal contraceptives [21]. Similarly, Satija described the psychosocial dimension of contraceptive choice, where fear, misinformation, and spousal opposition significantly shape attitudes and behaviors [22].

Cultural narratives continue to shape male involvement. Vasectomy acceptance remained poor in our cohort, especially in rural zones, echoing the findings of Oliveira et al., who reported that sterilization remains highly feminized due to myths surrounding male sterilization, such as perceived loss of strength or sexual performance [23]. Arora et al. similarly emphasized that female sterilization is often seen as the default owing to sociocultural conditioning, despite the availability of male options [24]. Additionally, health system barriers, such as the lack of provider counseling and the unavailability of preferred methods, were identified, particularly in the rural arm. Mahapatra et al. documented the role of community health workers and their insufficient training in vasectomy and newer contraceptive options, contributing to misinformation and method unavailability [25].

Another dimension revealed in this study was the impact of emotional well-being and self-efficacy on contraceptive decisions. Brault et al. found that sterilization, while irreversible, offered psychological relief to low-income urban women, granting them control over their fertility and improving marital dynamics [26]. This aligns with the low remission rates reported in our sterilized cohort. However, the sexual and reproductive health needs of young individuals remain inadequately addressed. Santhya and Jejeebhoy emphasized that young women face dual challenges-restricted autonomy and limited service availability-which are mirrored in our study, where younger participants (especially 18-25 years) showed lower contraceptive uptake, particularly in rural areas [27].

Our findings are consistent with those of Kunwar et al., who found that postpartum contraceptive uptake, although initially high, often drops due to misconceptions about fertility during lactational amenorrhea and postpartum amenorrhea [28]. The postpartum period remains an underutilized window for contraception counseling. Interestingly, contraceptive use before the first pregnancy was uncommon in the present study. Pandey and Singh documented similar trends, attributing this to cultural expectations around early childbearing and a lack of premarital contraceptive education in India [29]. This warrants a focus on educational interventions for married couples.

This study has a few limitations. The use of convenience sampling may introduce selection bias, limiting generalizability beyond the selected communities. Additionally, self-reported data on contraceptive use and decision-making may be subject to recall bias or social desirability bias. The exclusion of non-binary individuals and digitally illiterate participants may have omitted key subpopulations. Furthermore, the cross-sectional design limits causal interpretations, and the study did not capture changes in contraceptive behavior over time.

Future programs should focus on expanding method choice, particularly in rural and low-SES populations, through community-based awareness campaigns, male engagement, and strengthening frontline health worker training. Introducing youth-friendly services and promoting contraceptive education before marriage could improve early uptake. Health systems should invest in improving provider counseling, ensuring method availability, and reducing sociocultural barriers, particularly for male sterilization. Longitudinal research and deeper qualitative inquiry are warranted to explore motivations and long-term outcomes of contraceptive behaviors in different contexts.

## Conclusions

This study highlights significant disparities in contraceptive usage between urban and rural populations, driven by differences in education, socioeconomic status, decision-making autonomy, and access to reproductive health services. While urban participants showed a higher prevalence of modern reversible methods, rural populations predominantly relied on permanent methods such as sterilization. Factors such as literacy, self-decision-making, and urban residence were strongly associated with increased contraceptive use. The findings underscore the need for targeted, culturally sensitive interventions that expand method choice, promote male involvement, and strengthen health system capacity to address unmet contraceptive needs across diverse settings. Addressing these gaps is essential for advancing reproductive autonomy and improving family planning outcomes in India.

## Appendices

**Table 6:** Structured questionnaire for data collection of the study Credits: Logeswari B M and Niveditha Prasath.

Section	Item	Response Options / Fields
Participant ID	________________________	
II. PERSONAL DETAILS		
1	Age at Marriage	________ years
2	Duration of Marriage	________ years
3	Age at First Pregnancy	________ years
4	Active Married Life	☐ Yes ☐ No
5	Comorbidity	☐ Hypertension ☐ Diabetes ☐ Both ☐ None ☐Other: ________
6	Significant Surgical History	☐ Yes ☐ No If yes, specify: __________________
7	Weight	________ kg
8	Blood Pressure	________ mmHg
9	Hemoglobin (Hb)	________ g/dL
10	BSL - Fasting (if done)	________ mg/dL
11	BSL - Postprandial (if done)	________ mg/dL
12	BSL - Random (if done)	________ mg/dL
III. DETAILS OF CONCEPTION		
13	Parity	________
14	Interval Between Marriage and First Childbirth	________ years
15	Number of Living Children	________
16	Age of Last Child	________ years
17	Desired Number of Children	________
IV. CONTRACEPTIVE DETAILS		
18	Currently Planning Pregnancy?	☐ Yes ☐ No
19	Currently Using Contraceptives?	☐ Yes ☐ No
20	Type of Contraceptive Used	☐ Barrier ☐ OCP ☐ Injectable ☐ Implants ☐Permanent (Tubal/Vasectomy) ☐ IUCD ☐ Others: ________
21	Duration of Contraceptive Use	________ months/years
22	Ever Used Emergency Contraceptives?	☐ Yes ☐ No
23	If Yes, Frequency of Use	☐ Once ☐ Occasionally ☐ Regularly
24	Decision Maker for Contraceptive Use	☐ Self ☐ Spouse ☐ Joint Decision

**Table 7:** Qualitative interview questionnaire Credits: Ishita Rathore and Logeswari B M.

Q. No.	Interview Question
1	Who usually decides which contraceptive method is used in your family?
2	Have you ever disagreed with your spouse or family about using contraception?
3	Can you describe a time when someone else influenced your contraceptive decision?
4	Are there any religious beliefs in your family or community that affect the use of contraception?
5	How do people in your area talk about contraception—do they think it’s a personal choice, or something taboo?
6	What have you heard about contraceptives and how they work?
7	Who or what has been your main source of information about contraceptives? (e.g., ASHA, TV, family?)
8	Are there any side effects or dangers you’ve heard of that concern you?
9	Are contraceptives easily available in your area?
10	Have you ever stopped using a method because it wasn’t available at a nearby health center?
11	Is there a difference in availability between what’s offered in the city and in your village?
12	What role do men play in family planning in your family or community?
13	Have you or your partner ever discussed vasectomy or male methods?
14	Why do you think most contraceptive use is expected from women?
15	What kind of contraceptive methods do people around you prefer and why?
16	Are there any methods you avoid? Why?
17	What do people think about temporary vs permanent methods?
18	If you could change one thing about how contraception is discussed or provided in your area, what would it be?
19	What advice would you give to someone younger thinking about using contraception?
